# The New York Homœopathic Union

**Published:** 1898-01

**Authors:** 


### THE NEW YORK HOMŒOPATHIC UNION.

The regular monthly meeting of the New York Homœopathic Union was held at 62 West Forty-ninth Street, New York, Thursday, December 16th, 1897, the President, Edmund Carleton, M. D., in the chair. Owing to the unavoidable absence of the Secretary, Mr. W. S. Gould was chosen to act as Secretary *pro tempore*. Drs. Burnet, Clock, Finch, Franklin, Gillingham, Krause, Leverson, Pease, Rushmore, Talcott, and Vondergoltz were present; also a number of medical students. Drs. Woodward and Powel, being unable to attend, sent letters of regret; Drs. Fincke and O'Brien, in addition, sent papers.

It was moved by Dr. Finch that the meeting proceed to consider the subject selected for discussion—"Can Susceptibility to Climate be Cured?"—and that Dr. Fincke's communication be read. Motion seconded and carried unanimously. Dr. Fincke's paper was then read, as follows:

